# Vanderburgh County Medical Society

**Published:** 1880-05

**Authors:** 


### VANDERBURGH COUNTY MEDICAL SOCIETY.

Dr. W. S. Ross, a non-member of the Society, read a paper *On Non-Malignant Tumor of the Uterus.*—Some time since I was requested by Dr. Beard to accompany him and examine Mrs. S. who, the doctor thought, had a uterine tumor as large as a fœtal head. I, on examination, found the patient very anæmic from loss of blood from the uterus. She was 45 years of age, married, and the mother of one child, now 20 years old. She stated that she had been bleeding for many years, but could not state positively the exact length of time. Thought it was only the "turn of life."

During last spring while washing, having occasion to lift a tub full of water she strained herself to such an extent that something gave way and came down into the vagina. She said she thought her womb was coming out. On making a digital examination I found a large fibroid tumor—so large that it filled the entire pelvic cavity. By seperating the labia majora, I could distinctly see the tumor. It had very much the appearance of an inverted uterus. There was a very offensive sanious discharge from the vagina. By passing the finger up the right side of the tumor, I ascertained it to be of a polypoidal shape, and appeared to be quite as large as a fœtal head. The patient being very anxious for relief, readily assented to the operation of removal. On the 16th of November, 1879, in company with Drs. Bacon, Lining and Beard, the operation was preformed. We first placed the patient under the influence of chloroform; then, by the use of an ecraseur, armed with a piano wire, which being placed over the tumor in the shape of a loop, it was severed by the drawing up or tightning process. The tumor was so large that Dr. Bacon and myself made several unsuccessful efforts to remove it with the thumb and finger. Dr. Bacon then suggested the obstetrical forceps, which he used, and removed the tumor very successfully. Very little hemorrhage followed. The tumor, gentlemen, is before you. It weighs ten ounces, and is non-malignant. We applied sol. ferri sub-sulph. on cotton wool, to the stump, and directed the vagina to be well washed out with castile soap and water, twice daily; and, also, to use a solution of permanganate of potassa after each ablution.

The patient improved rapidly until the sixth day, when she attempted to get out of bed, and in so doing fell on the floor. The noise awoke her husband, who was sleeping in the room. She was lifted up and placed in bed. Since then she seems to have lost all will power. There was but little discharge from the vagina. Tongue broad, white and flabby. When asked to put her tongue out, she would roll it around in her mouth as if she had no control over it. I directed iron and quinine to be taken three times a day.

I lost sight of the patient (she being under the care of Dr. Beard) until several weeks had elapsed, when Dr. Bacon and myself called to see her—Dr. Beard having abandoned the case. We found her very anæmic, tongue broad, white and flabby; feet and legs swolen; bowels constipated; will-power still impaired. We made an examination and found considerable discharge from the uterus. The os was open and very much enlarged. We applied argent. nitris in stick, to the os, and carried it well up into the cervix. We also direct the following pill:

℞. *Aloes*.............................grs. x x
*Quinine*..........................grs. x x
*Ferri Sul Exc*......................grs. x x
*Nucis Vomicœe Ext*................grs. x x

M. Ft. pil. No. xx—sig. One, three times daily.

I saw the patient about ten days afterward. She had improved very much in general appearance. Her bowels were regular. The swelling had disappeared from her legs and feet. The tongue was very much improved in appearance; appetite good; no discharge from the vagina. The treatment was continued. The patient gradually improved, and is now enjoying reasonable health. I saw her yesterday.

Dr. Owens.: I have noticed, several months after an operation of this kind, that large granulations would appear on the stump and bleed profusely. I usually apply nitric acid to them, and they heal readily.

Dr. Day: There is no class of diseases that we should be more careful in prognosing than uterine tumor. I have had several cases come under my observation in which I made unfavorable prognoses, but they would seem to improve for a time, and think themselves cured. One case in particular: A lady came to me suffering from a malignant uterine tumor. I told her she could not expect to be cured, and that treatment was unnecessary. She put herself under somebody's treatment, however, I heard nothing more from her until I sent in my bill. She saw me not long afterward and scored me pretty roundly for frightening her and then sending in a bill for it.; yet, while she was talking to me I could see the cancerous cachexia in her features.

Dr. G. B. Walker: To give both sides of the question

I was called, in consultation with Dr. Day, a few years ago, to see a woman who had a uterine tumor. She was very much prostrated and apparently on the verge of death. We proceeded to operate, however, and removed the tumor. The patient made a good recovery, and, I think, is living to-day. I am in favor of giving these cases the benefits of an operation.

*Mysophobia—Pelvic Abscess.*—Dr. Irwin reported the following interesting case, which occurred in his practice several months ago: Mrs. S. came to see me, complaining of an irresistible desire to wash her hands and face. This had continued for eight months and was very annoying, having to wash the hands oftener at night than during the day and particularly when she was left alone. I made a vaginal examinatian and found an enlargement in Douglas' cul-de-sac, which I punctured. It proved to be a pelvic abscess, and discharged a large amount of greenish yellow pus. The os uteri was covered with large granulations. These I treated, and, in a short time the patient was entirely relieved of her uterine trouble and the desire to wash her hands (mysophobia) had almost left her.

*Acephalous Child—Miscarried after Eighth Month.*---Dr. G. B. Walker said, that he had recently met with a case of sufficient interest to justify its being reported. A lady about 25 years of age, pregnant with her second child, and a little more than 8 months gone, after a long ride was seized with violent pain, extending over the whole abdomen, but especially involving the uterus, intermittent in character, and evidently foreboding premature labor. By perseverance for 12 hours or more in rest and anodyne treatment, the pain abated, leaving general tenderness of the region implicated, which lasted for two or three days. Two weeks after this she was seized with regular labor-pains, commencing in the morning and continuing until said night, when the speaker was called to see her in consultation with Dr. Davidson, who had charge of the case. At this time the patient was somewhat exhausted, the pains growing weaker ; and no material change in the progress of labor had been noticed, for several hours. On examination it was found that the os uteri was dilated the size of a silver dollar and somewhat relaxed, with the membranse protruding one-third the distance between the upper and lower straits, the presentation, however, not being perceptible.

It was agreed to take the child away by the hand. In persuance of this determination, the hand was pushed through the membranes, followed by an immense discharge of amniotic fluid (hydramnios), something like a diminutive child's head was met with, which was readily grasped by the hand, and the child brought away. The placenta being loose in the uterine cavity, was shortly afterwards also taken away by the hand. It required some little attention to secure permanent contraction of the uterus, but otherwise no further accident occurred to the woman. The child was dead (had indeed not moved since the attack of pain two weeks before), it was rather below the medium size, and was acephalous. The upper part of the head was absent, or rather the cranium terminated just above the eyes and ears in front, and the occipital protuberance posteriorly; evidently the cerebrum was absent. The head, however, was well covered by scalp and hair and a bony vault. The whole head was no larger than a medium sized orange. Probably the same disease of the ovum which resulted in dropsy of the amnion, also prevented the full development of the brain.

No post-mortem examination was practicable.

